# Transcriptome Analysis of Watermelon Leaves Reveals Candidate Genes Responsive to *Cucumber green mottle mosaic virus* Infection

**DOI:** 10.3390/ijms20030610

**Published:** 2019-01-31

**Authors:** Yuyan Sun, Min Fan, Yanjun He

**Affiliations:** 1 Institute of Vegetables, Zhejiang Academy of Agricultural Sciences, Hangzhou 310021, China; syy1111@126.com (Y.S.); hyj1009@163.com (Y.H.); 2 Key Laboratory of Biology and Genetic Improvement of Horticultural Crops, Ministry of Agriculture, Beijing 100081, China

**Keywords:** watermelon, *Cucumber green mottle mosaic virus*, transcriptome, qRT-PCR

## Abstract

*Cucumber green mottle mosaic virus* (CGMMV) is a member of the genus *Tobamovirus*, which cause diseases in cucurbits, especially watermelon. In watermelon, symptoms develop on the whole plant, including leaves, stems, peduncles, and fruit. To better understand the molecular mechanisms of watermelon early responses to CGMMV infection, a comparative transcriptome analysis of 24 h CGMMV-infected and mock-inoculated watermelon leaves was performed. A total of 1641 differently expressed genes (DEGs) were identified, with 886 DEGs upregulated and 755 DEGs downregulated after CGMMV infection. A functional analysis indicated that the DEGs were involved in photosynthesis, plant–pathogen interactions, secondary metabolism, and plant hormone signal transduction. In addition, a few transcription factor families, including WRKY, MYB, HLH, bZIP and NAC, were responsive to the CGMMV-induced stress. To confirm the high-throughput sequencing results, 15 DEGs were validated by qRT-PCR analysis. The results provide insights into the identification of candidate genes or pathways involved in the responses of watermelon leaves to CGMMV infection.

## 1. Introduction

Watermelon [*Citrullus lanatus* (Thunb.) Matsum. & Nakai] is an important cucurbit crop grown worldwide. Watermelon fruit provide glucose, fructose, malic acid, lycopene, vitamins, and amino acids, which are desired by consumers. In 2016, approximately 117 million tons of watermelon were produced, accounting for 10.9% of total global vegetable production (available online: http://www.fao.org/faostat). China was the leading country, producing approximately 79 million tons of watermelon, which constituted 67.7% of the worldwide production (available online: http://www.fao.org/faostat). Currently, watermelon production is threatened by the *Cucumber green mottle mosaic virus* (CGMMV).

CGMMV is a member of the genus *Tobamovirus* in the Virgaviridae family. It has a 6.4-kb single-stranded, positive-sense RNA genome, which produces a stiff, rod-shaped 300 × 18-nm particle [1]. CGMMV is readily spread by contact, and pollen and seed transmission [2]. Since it was first report in 1935 in England, where it was found to cause diseases in cucumber [3,4], CGMMV has achieved a global distribution, being present in more than 30 countries, and has become a major threat to cucurbit-based industries [5,6,7,8]. In CGMMV-infected watermelon, mottling and mosaic develop on the leaves, brown necrotic lesions develop on the stems and peduncles, and the flesh becomes spongy and rotting, with dirty red discolorations [9]. 

Several investigations of plant–virus interactions have focused on transcriptional or post-transcriptional responses to CGMMV infections [10,11,12,13,14,15]. For instance, miRNA sequencing analysis of cucumber and watermelon leaves infected by CGMMV revealed potential miRNAs and target genes involved in responses to CGMMV-induced stress [10,14]. Profiles of CGMMV-derived siRNAs in infected cucumber [11], bottle gourd [12] and watermelon [15] indicated that vsiRNAs have complicated functions during host–virus interactions. In addition, transcriptome analysis of watermelon fruit responding to CGMMV-induced stress revealed that some differentially expressed genes (DEGs) related to cell wall components and photosynthesis may be directly involved in the formation of diseased watermelon symptoms [13]. However, gene expression levels in leaves of CGMMV-infected watermelons have not been studied. Here, we analyzed the early responses of watermelon leaves to CGMMV infection using RNA sequencing (RNA-Seq) and investigated alterations in gene expression levels between healthy and CGMMV-infected plants. A subset of DEGs involved in photosynthesis, plant–pathogen interactions, secondary metabolism and plant hormone signal transduction were identified. The results provide insights into the identification of candidate genes or pathways associated with responses of watermelon leaves to CGMMV infection.

## 2. Results

### 2.1. Phenotypes and Confirmation of CGMMV in Watermelon Post Inoculation

**Figure 1:**
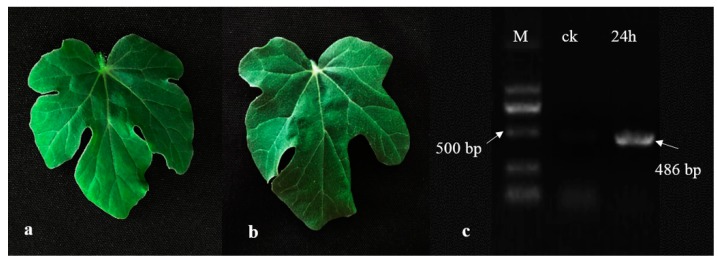
Phenotypes and confirmation of Cucumber green mottle mosaic virus (CGMMV) in watermelon leaves. (**a**) ck watermelon leaf; (**b**) 24-h post-inoculation (24 hpi) watermelon leaf; (**c**) RT-PCR confirmation of CGMMV accumulation in watermelon leaves.

At 24-h post-inoculation (24 hpi), there were no obvious disease symptoms exhibited in the watermelon leaves compared with the ck plants (Figure 1a,b). The accumulation of CGMMV in both ck and 24 hpi plants was confirmed by RT-PCR, the 24 hpi samples clearly amplified the specific 486-bp CGMMV CP fragment (Figure 1c). The results showed that even though no obvious symtoms developed in 24 hpi watermelon leaves, the virus has accumulated in the plants at the early infectious time. 

### 2.2. Overview of the RNA-Seq Results

Here, triplicates of treatments for ck and 24 hpi samples were conducted, and six leaf samples were prepared for RNA extraction and Illumina sequencing. A total of 282,952,260 raw reads were generated, encompassing 42.44 Gb data, which were sufficient for the gene expression analysis. After removing the low-quality reads and adapter sequences, 276,176,444 (97.60%) clean reads were obtained. Using TopHat2 software, 266,058,279 (96.33%) clean reads were mapped to the watermelon genome, and 200,211,479 (72.49%) unique reads were mapped the reference genome. The Q20 percentage was over 99.60%, and the Q30 percentage was over 96.06% (Table 1). 

### 2.3. Analysis of DEGs in Response to CGMMV-Induced Stress

**Figure 2:**
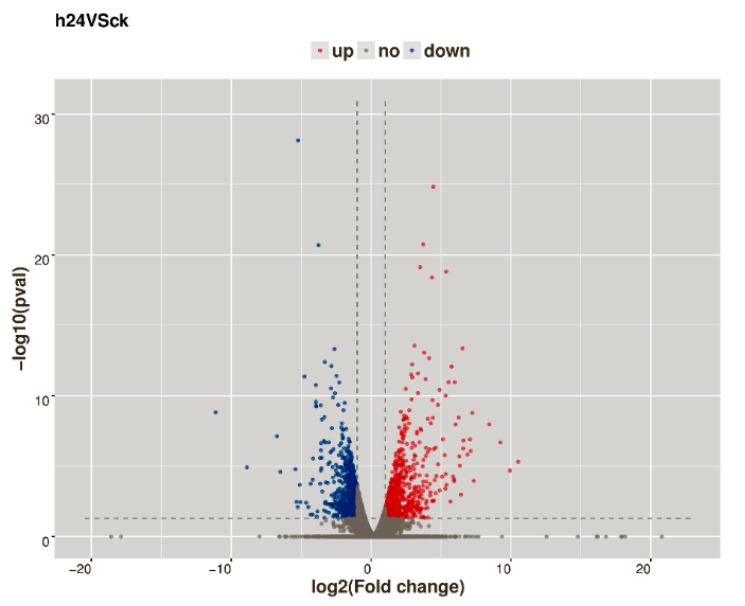
Volcano map representing the number of differentially expressed genes of watermelon leaf between the 24 hpi and ck. Red dots represent up-regulated genes, and green dots represent down-regulated genes.

**Figure 3:**
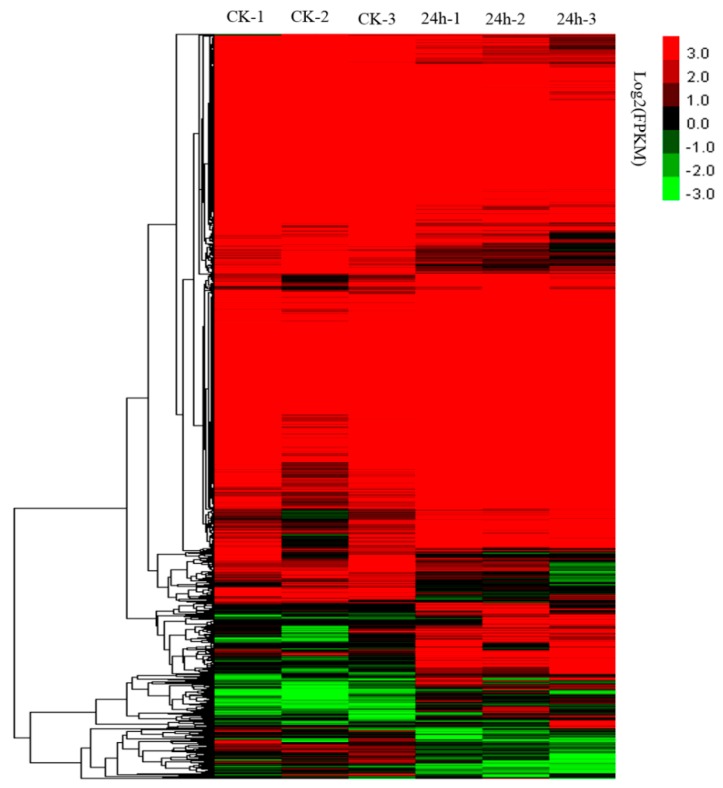
Hierarchical clustering of differential expression profiles for 1641 genes between the 24 hpi and ck watermelon leaf libraries (false discovery rate (FDR) ≥ 0.05 and absolute value of the log2 ratio > 1).

Genes with a false discovery rate (FDR) < 0.05 and an estimated absolute log2 fold change (log2FC) ≥ 1 in sequence counts between libraries were considered significantly differentially expressed. Finally, 1641 DEGs were identified between the 24 hpi and ck leaf libraries (Figure 2; Table S1). Of these genes, 886 were up-regulated and 755 were down-regulated after CGMMV infection. To provide an expression overview of the DEGs, hierarchical clustering was generated and is shown in Figure 3.

### 2.4. Gene Ontology (GO) and Kyoto Encyclopedia of Genes and Genomes (KEGG) Pathway Enrichment Analyses of DEGs

**Figure 4:**
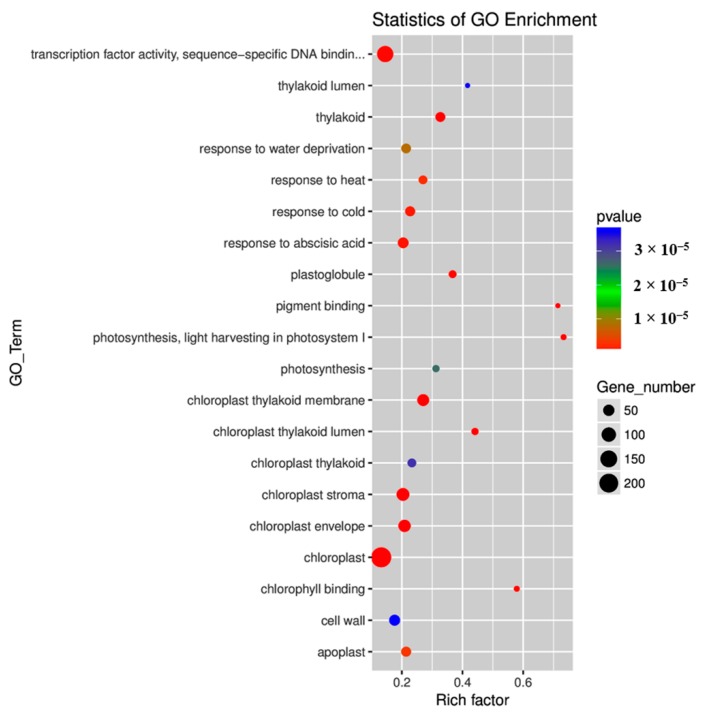
Top 20 gene ontology (GO) terms enriched for different expressed genes. The size of each circle represents the number of significantly differentially expressed genes enriched in the corresponding term. The enrichment factor was calculated using the number of enriched genes divided by the total number of background genes in the corresponding pathway. The *p*-value was calculated using the Benjamini–Hochberg correction. A term with *p* < 0.05 is considered significantly overrepresented.

On the basis of the GO classifications, DEGs were separated into three categories: molecular function, cellular component, and biological process (Table S2). In the category of molecular function, pigment binding, chlorophyll binding, transcription factor (TF) activity, and sequence-specific DNA binding were significantly enriched (Figure 4; Table S2). In the category of cellular component, chloroplast thylakoid membrane, thylakoid, chloroplast stroma, chloroplast envelope, chloroplast, chloroplast thylakoid lumen, plastoglobule, apoplast, chloroplast thylakoid, thylakoid lumen, and cell wall were significantly enriched (Figure 4; Table S2). In the category of biological process, photosynthesis, light harvesting in photosystem (PS) I, response to abscisic acid (ABA), response to cold, response to heat, response to water deprivation, and photosynthesis were significantly enriched (Figure 4; Table S2).

**Figure 5:**
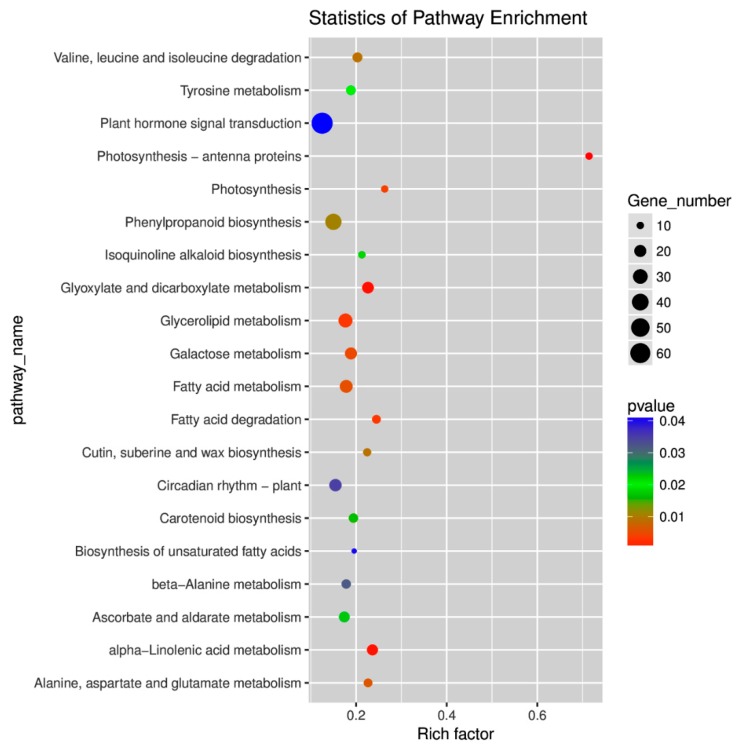
Statistics of the top 20 pathways enriched for differentially expressed genes. The size of each circle represents the number of significantly differentially expressed genes enriched in the corresponding pathway. The enrichment factor was calculated using the number of enriched genes divided by the total number of background genes in the corresponding pathway. The *p*-value was calculated using the Benjamini–Hochberg correction. A pathway with *p* < 0.05 is considered significantly overrepresented.

In addition, 122 KEGG pathways for the DEGs were identified in watermelon leaves under CGMMV-induced stress (Table S3). Among them, the plant hormone signal transduction pathway (68 DEGs) was the largest group, followed by the plant–pathogen interaction pathway (56 DEGs) and the carbon metabolic pathway (39 DEGs) (Figure 5; Table S3). The top 20 enrichment pathways (*p*-value < 0.05) were photosynthesis *p*-antenna proteins (ko00196), glyoxylate and dicarboxylate metabolism (ko00630), alpha-linolenic acid metabolism (ko00592), fatty acid degradation (ko00071), glycerolipid metabolism (ko00561), photosynthesis (ko00195), galactose metabolism (ko00052), fatty acid metabolism (ko01212), alanine, aspartate and glutamate metabolism (ko00250), valine, leucine and isoleucine degradation (ko00280), cutin, suberine, and wax biosynthesis (ko00073), phenylpropanoid biosynthesis (ko00940), carotenoid biosynthesis (ko00906), isoquinoline alkaloid biosynthesis (ko00950), tyrosine metabolism (ko00350), ascorbate and aldarate metabolism (ko00053), beta-alanine metabolism (ko00410), circadian rhythm–plant (ko04712), biosynthesis of unsaturated fatty acids (ko01040), and plant hormone signal transduction (ko04075) (Figure 5; Table S3). 

### 2.5. DEGs Involved in Photosynthesis

Photosynthesis, as the most fundamental and complex physiological process in plants, is disturbed by viral stress. Virus-infected plants are commonly characterized by decreased photosynthetic rates, which suggests that photosynthesis is a major activity suppressed by host antiviral defense responses [16,17]. In this study, 22 DEGs were associated with the pathway of photosynthesis (ko00195; ko00196) (Table 2). Among these, seven DEGs (Cla001715, Cla001790, Cla004698, Cla004699, Cla004704, Cla004703, and Cla007741), encoding the PsbQ, PsbO, PsbP, Psb27, and PsbW proteins, were involved in PSII. In addition, two DEGs (Cla007940 and Cla008429), encoding PSI reaction center subunits, were involved in PSI. Moreover, 10 DEGs (Cla004746, Cla011145, Cla011748, Cla012368, Cla013826, Cla018117, Cla019105, Cla019595, Cla022573, and Cla022963), encoding the chlorophyll a- and chlorophyll b-binding proteins, were involved in the light harvesting complex and serve as conduits of the excitation energy to the reaction center of PSII. All of these photosynthesis-related genes were down-regulated after CGMMV infection, suggesting a decreased photosynthetic efficiency upon CGMMV infection in the early stages.

### 2.6. DEGs Involved in Plant–Pathogen Interactions

Resistance to invading microorganisms is often governed by specific recognition between plant and pathogen proteins. In the present study, 55 DEGs (20 down- and 35 upregulated) were identified as participating in the pathway of plant–pathogen interaction (ko04626) (Table S4). These DEGs mainly encoded a receptor-like protein kinase, serine/threonine-protein kinase, disease resistance protein, calmodulin (CaM), CaM-like protein, and WRKY and MYB TFs. For example, DEGs encoding two CaMs (Cla003404 and Cla021803), one disease resistance protein (Cla020705), two WRKY TFs (Cla010918 and Cla014818), three MYB TFs (Cla005982, Cla015165, and Cla018610), 16 receptor-like protein kinases (Cla001490, Cla005698, Cla008301, Cla008723, Cla010837, Cla012567, Cla012568, Cla013903, Cla015191, Cla015419, Cla019852, Cla020279, Cla020764, Cla021452, Cla022195, and Cla022527) were upregulated after CGMMV infection. However, a few DEGs encoding receptor-like protein kinase, leucine-rich repeat-containing protein, and histone H2A were downregulated after CGMMV infection. These DEGs might play crucial roles in the responses to the CGMMV-induced stress.

### 2.7. DEGs Involved in Secondary Metabolism

Plant defense mechanisms against pathogens result in the differential regulation of various processes of primary and secondary metabolism. In the present study, a subset of DEGs involved in the pathways of secondary metabolism, such as phenylpropanoid biosynthesis (ko00940), carotenoid biosynthesis (ko00906), galactose metabolism (ko00052), and ascorbate and aldarate metabolism (ko00053), were significantly enriched (Table S5). 

Pathogen infection often activates the phenylpropanoid pathway, leading to the production of both phytoalexins and lignin/suberin precursors for cell wall strengthening [18]. In this study, 37 DEGs were associated with the pathway of phenylpropanoid biosynthesis. Among which, 26 DEGs, encoding proteins such as cinnamoyl-CoA reductase (Cla000214, Cla017205, Cla017206, and Cla018764), cinnamyl alcohol dehydrogenase (Cla016048), caffeoyl-CoA O-methyltransferase (Cla022971), 4-coumarate CoA ligase-like (Cla022956), peroxidase (Cla001825, Cla003187, Cla003188, Cla003189, Cla003191, Cla003194, Cla013410, Cla014249, Cla017829, and Cla018276), were up-regulated after CGMMV infection. In addition, 11 DEGs, encoding proteins such as phenylalanine ammonia-lyase (Cla018297 and Cla018301), GDSL esterase/lipase (Cla018383 and Cla019629), and 4-coumarate-CoA ligase (Cla006818), were downregulated under the CGMMV-induced stress.

Carotenoids or carotenoid catabolites can act as stress-signaling molecules [19]. In total, 13 DEGs were associated with the carotenoid biosynthetic pathway in this study, and seven, which encoded an F-box protein (Cla005171), GDSL esterase/lipase (Cla010854), cytochrome P450 (Cla015020, Cla016011, Cla020673, and Cla022574), and UDP glycosyltransferase (Cla016026), showed up-regulated trends after CGMMV-induced stress. In contrast, six DEGs, encoding a short-chain dehydrogenase/reductase (Cla001908), phytoene synthase (Cla005425), lycopene cyclase (Cla016840), cytochrome P450 (Cla005457), GDSL esterase/lipase (Cla007748), and beta-hydroxysteroid dehydrogenase (Cla018423), were down-regulated under CGMMV-induced stress.

Ascorbic acid accumulates as a defense response to *Turnip mosaic virus* in resistant *Brassica rapa* cultivars [20]. In this study, 17 DEGs were involved in the ascorbate and aldarate metabolic pathway, and six of them, encoding laccase (Cla006606 and Cla014164), aldehyde dehydrogenase (Cla015539 and Cla019804), inositol oxygenase 1 (Cla016814), and mono dehydroascorbate reductase (Cla017711), were up-regulated under the CGMMV-induced stress conditions. Furthermore, 11 DEGs, encoding laccase (Cla001819, Cla008184, Cla011558, Cla015786, and Cla022385), GDSL esterase/lipase (Cla012640 and Cla012639), ascorbate peroxidase (Cla013254), UDP-D-glucose dehydrogenase (Cla019072), inositol oxygenase 1 (Cla011244), and inducer of CBF expression 1 (Cla002645), were down-regulated under CGMMV-induced stress conditions.

In total, 20 DEGs associated with the galactose metabolic pathway were identified. Among them, ten DEGs, encoding beta-galactosidase (Cla012613, Cla020797, Cla011211, and Cla011212), stachyose synthase (Cla017113 and Cla015152), galactinol synthase (Cla009222), reductase 1 (Cla018029), zinc finger (Cla014795), and alkaline alpha galactosidase I (Cla023372), were down-regulated. However, the other ten DEGs, aldose 1-epimerase (Cla018918), galactinol synthase (Cla010080, Cla010955, and Cla017336), NADP-dependent sorbitol 6-phosphate dehydrogenase (Cla015879 and Cla015878), stachyose synthase (Cla003446), beta-fructofuranosidase insoluble isoenzyme 2 (Cla015574), phosphofructokinase (Cla004847), and zinc finger (Cla018432), were up-regulated after the CGMMV infection.

### 2.8. DEGs Involved in Plant Hormone Signal Transduction

During viral infections, the disruption of the plant’s normal developmental physiology has been associated with alterations in phytohormone accumulation and signaling [21]. In this study, a few DEGs were involved in the plant hormone signal transduction pathway (K04075), particularly the accumulation and signaling of auxin and ABA (Table S6). For example, the expression levels of six auxin-responsive proteins (Cla002975, Cla006021, Cla014808, Cla019806, Cla021571, and Cla022332) increased under the CGMMV-induced stress conditions, whereas the expression of another five auxin-responsive proteins (Cla005087, Cla012819, Cla015002, Cla017664, and Cla020817) decreased. In addition, an auxin transporter-like protein (Cla004339) showed a decreased expression pattern. In general, ABA is involved in the negative regulation of plant defenses against various biotrophic and necrotrophic pathogens. In this study, ABA insensitive (Cla017696) and ABA receptor (Cla015009) were down-regulated after CGMMV infection. MYC and MYB proteins function in an ABA-dependent manner [22]. In the present study, genes encoding MYB (Cla012305) and MYC-like protein (Cla006058) were downregulated, whereas a MYB-like protein gene (Cla020369) was upregulated under the CGMMV-induced stress conditions, functioning in an ABA-dependent manner. 

### 2.9. TFs Involved in CGMMV Stress Response

**Figure 6:**
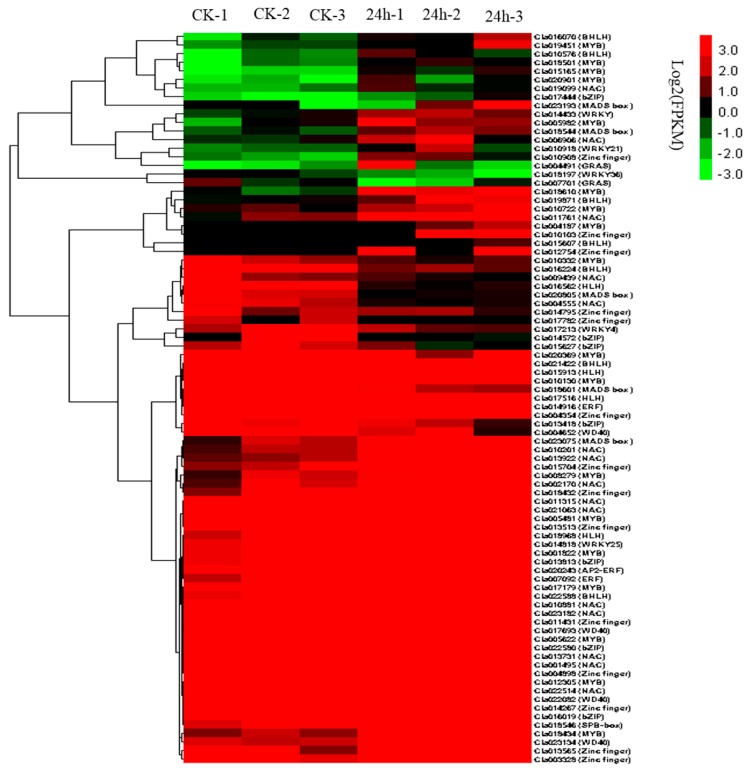
Transcription factors involved in responses to CGMMV infection in watermelon leaves.

The invasion of plant viruses induces the expression of a variety of genes, which are usually regulated by TFs [23]. In this study, we observed 85 TFs that were differentially expressed after CGMMV infection (Figure 6; Table S7). These TFs included MYB (18 genes), NAC (15 genes), zinc finger (14 genes), HLH (11 genes), BZIP (7 genes), WRKY (5 genes), MADS box (5 genes), WD-40 (4 genes), ERF (3 genes), GRAS (2 genes), and SBP-box (1 gene). The WRKY family members were mainly associated with the plant–pathogen interaction pathway. The GRAS and SBP-box members were mainly involved in the plant hormone signal transduction pathway. The MYB family members were mainly involved in the plant hormone signal transduction, plant–pathogen interaction, oxidative phosphorylation, and RNA transport pathways. The HLH and bZIP family members were mainly involved in the plant hormone signal transduction and protein processing in the endoplasmic reticulum pathways. The NAC family members were involved in RNA degradation and glycerolipid metabolic pathways. The MADS box family members were involved in the RNA transport and protein export pathways. The WD-40 genes were involved in the ubiquitin-mediated proteolysis and ribosome biogenesis in eukaryotes pathways. The zinc finger genes were involved in the galactose metabolism, circadian rhythm, RNA transport, and ribosome pathways. 

### 2.10. qRT-PCR Validation of DEGs

**Figure 7:**
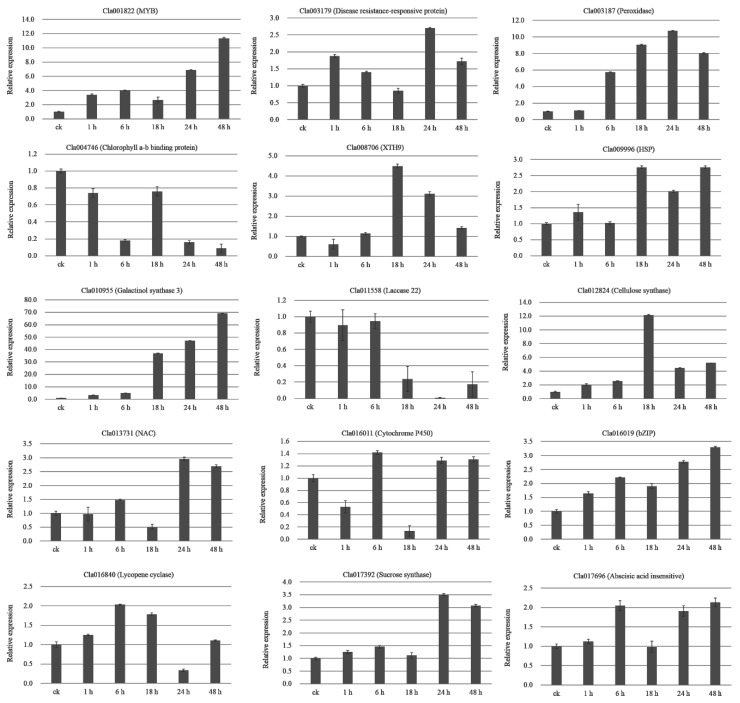
qRT-PCR analysis of the expression of CGMMV response-related genes. The *y*-axis represents the log2 values (CGMMV/ck). Analyses were completed in triplicate, and the error bars represent the standard errors.

In total, 15 DEGs were selected for qRT-PCR validation (Figure 7). Cla010955 (galactinol synthase 3) was gradually up-regulated during the CGMMV-infection process. Cla001822 (MYB), Cla016019 (bZIP), Cla016840 (lycopene cyclase), and Cla017696 (ABA insensitive) were continually up-regulated at 0- (control, ck), 1-, and 6-h, slightly down-regulated at 18-h, and up-regulated again at 24- and 48-h post-CGMMV infection. The expression of Cla004746 (Chlorophyll a-b binding protein) decreased at ck, 1-, and 6-h, sharply increased at 18-h, and then decreased again at 24- and 48-h post-CGMMV infection. Cla003187 (peroxidase) was continually up-regulated at ck, 1-, 6-, 18-, and 24-h and then, was down-regulated at 48-h post-CGMMV infection. The expression patterns of Cla013731 (NAC) and Cla017392 (sucrose synthase) were similar, remaining relatively stably expressed at ck, 1-, and 6-h. They were then slightly down-regulated at 18-h and up-regulated at 24- and 48-h post-CGMMV infection. Cla008706 (XTH9) remained at a relatively stable expression level at ck, 1-, and 6-h, was sharply up-regulated at 18-h and then down-regulated at 24- and 48-h post-CGMMV infection. Cla009996 (HSP) remained relatively stably expressed at ck, 1-, and 6-h post-CGMMV infection. It was then sharply upregulated at 18-h, downregulated at 24-h, and upregulated again at 48-h post-CGMMV infection. Cla009996 (disease resistance-responsive protein) was upregulated at 1-h, downregulated at 6- and 18-h, sharply upregulated at 24-h and downregulated at 48-h post-CGMMV infection. Cla011558 (laccase 22) remained at a stable expression level at ck, 1-, and 6-h, decreased at 18- and 24-h, and was then upregulated at 48-h post-CGMMV infection. The expression of Cla012824 (cellulose synthase) was stable at ck, 1- and 6-h, increased at 18-h, and then decreased to a relatively stable level at 24- and 48-h post-CGMMV infection. Cla016011 (cytochrome P450) was downregulated at 1-h, upregulated at 6-h, then downregulated at 18-h, and upregulated at 24- and 48-h post-CGMMV infection. The qRT-PCR data were generally consistent with the high-throughput transcriptome results, indicating that the transcriptome sequencing data were reliable. 

## 3. Discussion

First described in 1935 as infecting cucumber, CGMMV was one of the first plant viruses to be studied [4]. In CGMMV-infected watermelon, mottling and mosaicism develop on the leaves, brown necrotic lesions develop on the stems, and sponginess, rotting, and discolorations develop on the flesh. In addition to causing marketable yield losses owing to poor fruit quality, CGMMV also causes gross yield losses of > 50% in watermelon [24]. High-throughput sequencing technologies, such as RNA-Seq, are widely used to investigate the transcriptional profiles of different plants under virus-induced stress conditions [25,26]. RNA-Seq has also been utilized to identify the transcriptomic changes that occur during CGMMV-induced stress in bottle gourd leaves and fruit [27] and in watermelon fruit [13]. However, at present, there are no reports regarding the transcriptome early responses of watermelon leaves to CGMMV infection. Here, we performed transcriptome sequencing to assess the differences in gene expressions in the leaves of watermelon experiencing 24 h CGMMV-induced stress. The transcriptome data presented in this study provide insights into the early responses of watermelon leaves to CGMMV-induced stress and help us mine candidate genes or pathways to further illuminate the molecular mechanisms of watermelon responses to CGMMV-induced stress.

### 3.1. DEGs in Watermelon Leaves and Fruit

Under CGMMV-induced stress conditions, 1641 DEGs were identified in watermelon leaves, with 886 DEGs (53.99%) being upregulated and 755 DEGs (46.01%) being downregulated (Figure 3 and Table S1). These DEGs were mainly involved in photosynthesis, plant–pathogen interactions, secondary metabolism (phenylpropanoid biosynthesis, carotenoid biosynthesis, ascorbate and aldarate metabolism, and galactose metabolism), and plant hormone signal transduction pathways (Table S3). In a previous study, transcriptome profiles of CGMMV-infected and mock-inoculated watermelon fruit revealed 1621 DEGs, with 1052 up-regulated and 569 downregulated [13]. The DEG numbers in watermelon fruit was similar to the numbers in watermelon leaves. Consistent with our results, most of the DEGs in watermelon fruit were involved in metabolic processes, signal transduction, and plant–pathogen interactions [13]. Thus, common pathways were induced in the watermelon fruit and leaves by CGMMV infections. 

### 3.2. Changes in Photosynthesis After CGMMV Infection

Changes in photosynthetic pathways after pathogen infection have been reported earlier [16,17]. Switching on the defense mechanisms and respiratory processes is a cost-intensive process, which might occur at the expense of photosynthesis [16,17]. In this study, 22 DEGs involved in the photosynthetic pathway were completely downregulated after CGMMV infection (Table 2). These DEGs encoded PsbO, PsbP, PsbQ, Psb27, PsbW, PSI reaction center subunits, and chlorophyll a- and b-binding proteins. The PSII of oxygenic organisms is a multi-subunit pigment–protein complex that catalyzes the photo-oxidation of water with a concomitant reduction of the catalysis of plastoquinone to plastoquinol [28]. PsbO, PsbP, and PsbQ proteins may perform alternative functions within PSII, and all three of the extrinsic proteins were required for the accumulation of PSII reaction centers in higher plants [29]. The PsbO component may exhibit carbonic anhydrase [30] and/or GTPase activity [31], the PsbP protein may be involved in binding the manganese required for photoactivation [32] and, along with PsbQ, participate in granal stack formation [33]. PsbW, a 6.1-kDa low-molecular-weight protein, is located close to the minor antenna of the PSII complex and is important for the contact and stability between several PSII–light harvesting complex II supercomplexes [34]. Psb27 plays a fundamental role in enabling plants to adapt to changes in light intensity independently of the formation of PSII supercomplexes and state transitions [35]. Chlorophyll a- and b-binding proteins are involved in the light harvesting complex and serve as conduits of excitation energy to the reaction center of PSII [36]. These reduced photosynthetic gene activity levels might be associated with chlorosis or decreased green-tissue surface areas after the systemic spread of a virus.

### 3.3. Changes in Plant–Pathogen Interactions After CGMMV Infection

Plants have evolved innate immune systems that recognize the presence of pathogens and initiate effective defense responses, whereas pathogens have evolved effector proteins that can suppress host immune responses. These form the so-called plant–pathogen interactions [37]. In the present study, 55 DEGs encoding receptor-like protein kinase, CaM-like protein, CaM, serine/threonine-protein kinase, and WRKY and MYB TFs were involved in the plant–pathogen interaction pathway (Table S4). 

In response to several biotic and abiotic stresses, CaM plays an important role in sensing and transducing changes in cellular Ca^2+^ concentrations [38]. Several studies implicate CaMs in plant defense [39,40]. Transgenic tobacco (*Nicotiana benthamiana*) plants over-expressing *SCaM4* and *SCaM5* showed spontaneous lesions, increased PR genes expression and enhanced resistance to pathogens. The expression of tobacco *NtCaM13*, which is closely related to *SCaM4* and *SCaM5*, was elevated at the RNA and protein levels in TMV-infected leaves [40]. In this study, two genes encoding CaMs were upregulated after the CGMMV infection, indicating these two *CaMs* were induced by the presence of CGMMV.

Receptor-like kinases/proteins (RLKs/RLPs) are on the front lines of the battle between plants and pathogens, because they are present at the plasma membrane and perceive signature molecules from either the invading pathogen or damaged plant tissue. These RLKs and RLPs perceive PAMPs from pathogens and initiate PAMP-triggered immunity, the first layer of plant innate immunity [41]. *NbLRK1*, a lectin RLK found in *N. benthamiana*, was identified as an interactor of the protein INF1, an elicitin from *Phytophthora infestans* [42]. Another RLK, was discovered in *Nicotiana glutinosa* and interacts directly with the fungal elicitin capsicein from *Phytophthora capsici* [43]. In this study, 26 RLKs were identified, with 16 upregulated and 10 downregulated after CGMMV infection. These RLKs might play crucial roles in watermelon’s innate immunity. 

### 3.4. The Main TF Families Responding to CGMMV-Induced Stress

Briefly, TFs are composed of a DNA-binding domain that interacts with the cis-regulatory elements of its target genes and a protein–protein interaction domain that facilitates oligomerization between TFs and other regulators [44]. CGMMV-induced stress triggers extensive alterations in plant transcriptomes [13,27]. In a previous report, TFs, including those of the WRKY, MYB, bHLH, DREB, and ERF families, were significantly affected in watermelon fruit [13]. In our study, 85 differentially expressed TFs were identified in watermelon leaves after CGMMV infection (Figure 6; Table S7). The TF families identified in this study included MYB (18), NAC (15), zinc finger (14), HLH (11), BZIP (7), WRKY (5), MADS box (5), WD-40 (4), ERF (3), GRAS (2), and SBP-box (1). These TF families play vital roles in the transcriptional regulation of plants. 

The plant-specific WRKY TFs form one of the largest TF families, and they are a class of DNA-binding proteins primarily found in plants. The WRKY domain contains approximately 60 amino acids, comprising a highly conserved short peptide, WRKYGQK, at the N-terminus and a C2H2 or C2HC zinc-binding motif at the C-terminus [45]. WRKYs play important roles in plant–pathogen interactions. WRKY27, a WRKY protein from pepper, positively regulates the stress-resistance response to a *Ralstonia solanacearum* infection by modulating SA-, JA- and ET-mediated signaling pathways in tobacco [46]. In tomato, six WRKY Group III TFs were identified, and these TFs respond to TYLCV infection [47]. In this study, we observed that two WRKY TFs (Cla017213 and Cla018197) were down-regulated and three WRKY TFs (Cla014433, Cla014818, and Cla010918) were up-regulated in the leaf tissues of watermelon after CGMMV infection (Figure 6; Table S7). Thus, WRKY TFs are also involved in CGMMV-induced stress responses.

NAC TFs belong to plant-specific TF family, and they have been identified in many plant species. The NAC protein contains a highly conserved DNA-binding domain at the N-terminus and diverse transcription activation or repression domains at the C-terminus [48,49]. In plants, the NAC TFs act as nodes of a regulatory network that responds to biotic stresses [50,51]. NAC TFs also take part in plant–pathogen interactions. Two wheat NAC TFs (*TaNAC4* and *TaNAC8*) act as transcriptional activators in defense responses against strip rust pathogen infection [52,53]. The *Arabidopsis* NAC083 protein interacts with the Rep protein of *Mungbean yellow mosaic India virus* and plays important roles in viral DNA replication [51]. In tomato, six NAC TFs involved in the response to TYLCV infection were identified in resistant and susceptible cultivars [54]. In our study, 15 NAC TFs were affected by CGMMV infection in watermelon leaves (Figure 6; Table S7). Among them, 13 and 2 NAC TFs were up- and downregulated, respectively. The upregulated NAC TFs in leaf tissues might be involved in the adaptation to CGMMV-induced stress.

The MYB TFs are characterized by a DNA-binding MYB domain. The MYB domain is composed of approximately 52 amino acid residues that adopt a helix–turn–helix conformation that intercalates into the major groove of DNA [55,56]. After the first plant MYB gene, COLORED1, which is involved in anthocyanin biosynthesis, was identified in maize [57], a large number of MYB proteins were identified in different plant species. For example, in *Arabidopsis,* BOTRYTIS-SUSCEPTIBLE1, an R2R3-MYB gene (AtMYB108), is required for restricting the spread of two necrotrophic pathogens, *Botrytis cinerea* and *Alternaria brassicicola*, and is involved in the tolerance to osmotic and oxidative stresses [58]. In barley, the MYB TF *MYB6* functions as positive regulator of basal and MLA-mediated immunity responses to *Blumeria graminis* [59]. In this study, we observed that 3 MYB TFs were down-regulated and 15 MYB TFs were up-regulated in the leaf tissues of watermelon after CGMMV infection (Figure 6; Table S7). In addition, we compared these MYB TFs in watermelon leaves with those in watermelon fruit. A MYB gene, Cla017179, was commonly up-regulated in both leaves and fruit after CGMMV infection, indicating the important role of Cla017179 in responses to CGMMV infection.

The bZIP TFs are vital players in plant innate immunity owing to their ability to regulate genes associated with PAMP-triggered immunity, effector-triggered immunity, and hormonal signaling networks [60,61]. One bZIP member, G/HBF-1 from *Glycine max*, binds to G-box and H-box motifs that have strong links to pathogen elicitors. These motifs have also been reported in the promoter of the *chs15* gene, which is involved in flavonoid production in response to pathogen infection [62]. In this study, we observed that three bZIP TFs were down-regulated and four bZIP TFs were up-regulated in the leaf tissues of watermelon after CGMMV infection (Figure 6; Table S7). These bZIPs in watermelon act as positive or negative regulators and mediate the responses to CGMMV infection. 

## 4. Materials and Methods

### 4.1. Plant Materials and the CGMMV Treatment

The seeds of the watermelon advanced inbred line ‘JJZ-M’, which is susceptive to CGMMV, were planted in a greenhouse and maintained at 25 °C. Seedlings at the two-true leaf stage were inoculated with the CGMMV virus. Mock plants were inoculated with sodium phosphate buffer (pH 7.2). Leaves were harvested at 24-h post-inoculation (24 hpi). The presence of CGMMV was confirmed by RT-PCR using CGMMV coat protein specific primers (F: 5′-ATGGCTTACAATCCGATCACAC-3′; R: 5′-CTAAGCTTTCGAGGTGGTAGCC-3′). Leaves from three plants were mixed to form a biological replicate, and three biological replicates for each treatment were used for the high-throughput RNA-Seq and analysis.

### 4.2. mRNA Library Construction and Sequencing

Total RNA was extracted using TRIzol reagent (Invitrogen, Carlsbad, CA, USA) following the manufacturer’s procedure. RNA quantity and purity were analyzed using a Bioanalyzer 2100 and RNA 6000 Nano Lab Chip Kit (Agilent, Santa Clara, CA, USA) with RIN number >7.0. Approximately 10 µg total RNA, representing a specific adipose type, was subjected to Poly(A) mRNA isolation using poly-T oligo-attached magnetic beads (Invitrogen, Carlsbad, CA, USA). Following purification, the mRNA was fragmented into small pieces using divalent cations under an elevated temperature. Then, the cleaved RNA fragments were reverse-transcribed to create the final cDNA library in accordance with the protocol for the mRNA-Seq sample preparation kit (Illumina, San Diego, CA, USA). The average insert size for the paired-end libraries was 300 bp (± 50 bp). Then, we performed the paired-end sequencing on an Illumina HiSeq 4000 at LC Sciences (Houston, TX, USA) following the vendor’s recommended protocol. Prior to assembly, the low-quality reads ((1) reads containing sequencing adaptors; (2) reads containing sequencing primers; and (3) nucleotides with q quality scores lower than 20) were removed. The raw sequence data was submitted to the NCBI Short Read Archive.

### 4.3. RNA-Seq Read Mapping

We aligned reads to the watermelon genome [63] using hierarchical indexing for spliced alignment of transcripts (HISAT) [64], which initially removed a portion of the reads based on the quality information accompanying each read and then mapped the reads to the reference genome. HISAT allowed multiple alignments per read (up to 20 by default) and a maximum of two mismatches when mapping the reads to the watermelon genome. 

### 4.4. Transcript Abundance Estimation and Differential Expression Analysis

The mapped reads of each sample were assembled using StringTie [65]. Then, the transcriptomes from all the samples were merged to reconstruct a comprehensive transcriptome using perl scripts. After the final transcriptome was generated, StringTie and Ballgown [66] were used to estimate the expression levels of the transcripts. StringTie was used to determine expression levels for mRNAs by calculating fragments per kilobase of exon per million reads [67]. 

A differential expression analysis between the treatments (three biological replicates per treatment) was performed using the DESeq R package. DESeq provides statistical methods for determining differential expression in digital gene expression data using a model based on the negative binomial distribution. The differentially expressed mRNAs and genes were determined with log2 (fold change) > 1 or log2 (fold change) ≤ 1 and with statistical significance (*p*-value < 0.05) using the Ballgown R package. 

### 4.5. GO and KEGG Enrichment Analyses of DEGs 

The GO enrichment analysis of DEGs was performed using the GOseq R package in which gene length bias was corrected. GO terms with corrected *p* values < 0.05 were considered significantly enriched by DEGs. The KEGG database aids in understanding high-level functions and utilities of biological systems, such as the cell, organism and ecosystem, using molecular-level information, particularly large-scale datasets generated by genome sequencing and other high-throughput experimental technologies. We used KOBAS software to test the statistical enrichment of DEGs in KEGG pathways.

### 4.6. Validation of RNA-Seq Gene Expression Using qRT-PCR

To validate the gene expression levels revealed by the transcriptome data, leaves of watermelon plants were collected at 0-, 1-, 6-, 12-, 24- and 48-h post-CGMMV inoculation and used for RNA extraction. Total RNA (~2 μg) was reverse transcribed into cDNA using the TransScript One-Step gDNA Removal and cDNA Synthesis SuperMix (TransGen Biotech, Beijing, China). cDNA was then used as the template for qRT-PCR, which was completed in a 20-μL sample containing 10 μL 2× TransStart Top Green qPCR SuperMix (TransGen Biotech, Beijing, China), 2.0 μL diluted cDNA, 0.4 μL 50× Passive Reference Dye I, and 0.4 μL each primer (10 mM). qRT-PCR was conducted using StepOne Real-Time PCR System (ABI, Foster City, CA, USA) with the following program: 95 °C for 30 s, 40 cycles of 95 °C for 5 s, 55 °C for 15 s, and 72 °C for 10 s. Each experiment was run in triplicate. The relative expression levels were calculated based on the expression of the housekeeping Tubulin gene. The fold changes in gene expression were estimated in terms of threshold cycles using the 2^−∆∆*C*T^ method [68]. The primers designed for use in the qRT-PCR analysis are listed in Table S8. 

## 5. Conclusions

In this study, we comparatively analyzed the gene expression profiles of 24 hpi and ck watermelon leaves. The CGMMV infection affected the gene expression levels of 1641 DEGs, with 886 upregulated and 755 downregulated. A functional analysis of DEGs based on a GO annotation and KEGG pathway analysis showed that these DEGs were mainly involved in photosynthesis, plant–pathogen interactions, secondary metabolism, and plant hormone signal transduction. The CGMMV infection also affected the expression levels of several TF families, including WRKY, NAC, MYB, bZIP, and zinc finger. Our genome-wide transcriptome analysis provides a basis for the further investigation of the molecular mechanisms underlying CGMMV infection in watermelon. 

## Figures and Tables

**Table 1:** Overview of the watermelon leaf RNA sequencing (RNA-Seq) data.

Sample	Raw Reads	Raw Base	Clean Reads	Mapped Reads	Unique Mapped Reads	Q20%	Q30%
ck_1	52,659,470	7.90 G	52,037,998 (98.82%)	50,088,200 (96.25%)	38,860,793 (74.68%)	99.75	96.64
ck_2	49,566,772	7.44 G	49,101,272 (99.06%)	46,572,421 (94.85%)	32,009,543 (65.19%)	99.67	96.41
ck_3	43,812,198	6.57 G	43,327,768 (98.89%)	41,915,065 (96.74%)	32,382,435 (74.74%)	99.71	96.34
24h_1	41,190,724	6.18 G	36,924,504 (89.64%)	35,371,699 (95.79%)	27,425,778 (74.28%)	99.60	96.56
24h_2	46,758,132	7.01 G	46,328,460 (99.08%)	44,965,010 (97.06%)	33,083,469 (71.41%)	99.83	97.30
24h_3	48,964,964	7.34 G	48,456,442 (98.96%)	47,145,884 (97.30%)	36,449,461 (75.22%)	99.71	96.06

**Table 2:** Differentially expressed genes in watermelon leaves involved in photosynthesis.

Gene Name	Description	ck_1	ck_2	ck_3	24h_1	24h_2	24h_3	log2(fc)	Regulation
Cla000152	Sex-linked protein 9 (Fragment)	275.6	343.6	292.0	165.5	117.5	124.1	−1.16	down
Cla001715	PsbQ	23.4	41.5	27.0	17.7	13.2	10.5	−1.15	down
Cla001790	Oxygen-evolving enhancer protein 1 of photosystem II	223.6	66.0	231.7	25.5	20.8	144.1	−1.45	down
Cla004698	PsbP	56.5	98.3	56.1	26.9	25.5	22.6	−1.49	down
Cla004704	PsbP	47.6	60.1	44.4	26.3	17.6	21.4	−1.22	down
Cla004703	PsbP	47.6	60.1	44.4	26.3	17.6	21.4	−1.22	down
Cla007741	Photosystem II protein Psb27	33.5	40.9	37.7	20.2	12.0	13.7	−1.29	down
Cla007940	Photosystem I reaction center subunit XI	13.9	17.2	15.0	6.4	6.5	7.3	−1.19	down
Cla008429	Photosystem I reaction center subunit N	230.9	231.3	221.6	128.3	92.9	105.8	−1.06	down
Cla008898	Ferredoxin--NADP reductase	607.9	870.4	595.8	381.3	298.7	291.4	−1.09	down
Cla019799	Photosystem II reaction center W protein	25.1	11.2	12.5	4.7	7.2	8.2	−1.27	down
Cla019798	Photosystem II reaction center W protein	25.1	11.2	12.5	4.7	7.2	8.2	−1.27	down
Cla004746	Chlorophyll a-b binding protein 6A	308.0	547.5	409.9	201.0	146.4	115.9	−1.45	down
Cla011145	Chlorophyll a-b binding protein	92.2	154.3	87.8	47.6	34.8	51.2	−1.32	down
Cla011748	Chlorophyll a-b binding protein 13	192.7	320.0	218.0	152.7	105.8	74.4	−1.13	down
Cla012368	Chlorophyll a-b binding protein 8	733.7	1294.7	799.6	545.9	441.2	373.9	−1.06	down
Cla013826	Chlorophyll a-b binding protein	208.0	246.4	260.1	90.2	85.7	95.0	−1.40	down
Cla018117	Chlorophyll a-b binding protein 6	34.1	31.5	36.9	13.9	11.8	15.4	−1.32	down
Cla019105	Chlorophyll a-b binding protein P4	903.5	1298.7	1142.1	647.1	447.7	408.2	−1.15	down
Cla019595	Chlorophyll a-b binding protein 21	4.7	20.2	6.3	4.2	1.6	2.5	−1.89	down
Cla022573	Chlorophyll a-b binding protein 4	21.1	41.4	26.2	10.8	8.9	10.0	−1.58	down
Cla022963	Chlorophyll a-b binding protein 7	1106.7	1615.0	1306.7	786.1	563.1	628.6	−1.03	down

## Supplementary Materials

Supplementary materials can be found at http://www.mdpi.com/1422-0067/20/3/610/s1. Table S1. Genes differentially expressed between mock- and CGMMV-infected watermelon leaf libraries. Table S2. GO term enrichment for differentially expressed genes. Table S3. KEGG pathway enrichment for differentially expressed genes. Table S4. Differentially expressed genes involved in the plant–pathogen interaction pathway. Table S5. Differentially expressed genes involved in the secondary biosynthetic pathway. Table S6. Differentially expressed genes involved in the plant hormone and signal transduction pathway. Table S7. Transcription factors involved in responses to CGMMV infection in watermelon leaves. Table S8. Primers used for qRT-PCR validation.

## Availability of Sequencing Data

Sequencing data from this article were deposited at the sequence read archive (SRA) of the National Center for Biotechnology Information (NCBI) under the accession number of PRJNA514378.
